# The influence of sagittal deformity of the distal radius on pronosupination: a cadaver study

**DOI:** 10.1177/17531934221117448

**Published:** 2022-09-13

**Authors:** Lucille Auberson, Jean-Yves Beaulieu, Lionel Athlani

**Affiliations:** 1 Department of Hand Surgery, Division of Orthopaedics and Trauma Surgery, Geneva University Hospitals, Geneva, Switzerland; 2 Department of Anatomy, Faculty of Medicine, University of Geneva, Geneva, Switzerland; 3 Department of Hand Surgery, Plastic and Reconstructive Surgery, Centre Chirurgical Emile Gallé, CHU de Nancy, Nancy, France

**Keywords:** Malunion, pronation, radius, sagittal deformity, supination, anterior plate

## Abstract

We performed a cadaver study using seven fresh-frozen adult upper limbs to assess the impact of increasingly larger distal radial deformity in the sagittal plane on the range of motion in pronation/supination. Three palmar (20°, 30° and 40° angulations) and three dorsal (10°, 20° and 30° angulations) tilt deformities, without any radial shortening, were simulated by performing a radial osteotomy and using custom-made three-dimensional-printed anterior plates. We measured the maximum unconstrained pronation and supination before the osteotomy and after each induced deformation. There was a decrease in the median pronation and supination values for all palmar and dorsal tilt deformities. The pronation range was more impaired than the supination range, and dorsal tilt deformities caused the greatest loss in forearm rotation. Our results suggest that forearm rotation in both pronation and supination is reduced as soon as 10° to 20° distal radial deformity occurs in the palmar or dorsal direction.

## Introduction

Forearm rotation is essential for carrying out activities of daily living by allowing a multitude of complex hand positions (Tynan et al., 2000).

In a cadaver study, Bronstein et al. (2014) indicated that a radial shortening of 10 mm reduced forearm pronation by 47% and supination by 29%, whereas 30° dorsal tilt did not significantly restrict the range of motion. Pogue et al. (1990) emphasized the importance of radial shortening in reducing pronation and supination but minimizing the impact of sagittal deformity. We hypothesized that a palmar or dorsal tilt deformity, even if minor, may reduce pronation and supination.

In this cadaver study, we assessed the impact of increasingly larger distal radial deformity in the sagittal plane on the range of motion of pronation and supination.

## Methods

In this cadaver study, we studied seven fresh-frozen, unembalmed adult upper limbs. Two right and five left limbs were previously amputated at the mid humerus level and prepared using the same protocol by one specialist surgeon of Level IV experience (Tang and Giddins, 2016). None of the specimens had any scars on the hand, wrist and forearm and to our knowledge, none had any previous history of hand and wrist pathology, including fractures or lacerations. The age of the cadavers ranged from 56 to 82 years, with an average of 72 years. There were five males and two females.

### Preparation of the cadavers for testing

**Figure 1.**
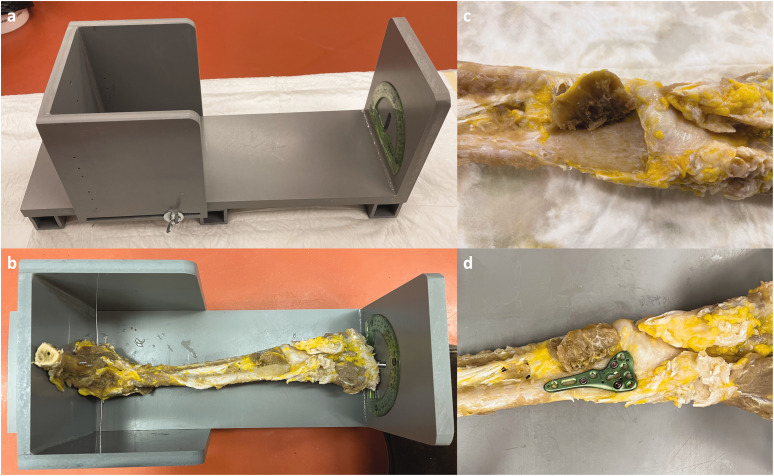
Experimental set-up. (a) The custom-made jig can be adjusted to the forearm's size and has a goniometer for measuring the pronation and supination amplitudes. (b) The prepared specimen was installed in the jig with the elbow secured in 90° flexion with K-wires. (c) Pronator quadratus muscle elevated and (d) anterior plate secured with two epiphyseal and two diaphyseal screws positioned in predefined pilot holes.

First, the skin, subcutaneous tissue and muscles were removed, preserving only the intrinsic and extrinsic ligament structures of the elbow and wrist joints, the interosseous membrane and the pronator quadratus. Next, all the digits were amputated at the metacarpophalangeal joints. Elevation of the pronator quadratus exposed the distal radius to allow an anatomic locking anterior plate provided by Newclip Technics™ (left and right Xpert Wrist 2.4, PSI radius division, Haute-Goulaine, France) to be osteosynthesized to the bone. Finally, two epiphyseal and two diaphyseal screws were positioned at the predefined pilot holes. At this point, we used a C-arm to confirm the absence of pre-existing distal radial malunion and correct positioning of the plate and screws. The limb was then fixed to a specially designed jig with the elbow secured in 90° flexion and the forearm completely free for pronation and supination (Figure 1).

### Simulation of deformity in the sagittal plane

**Figure 2.**
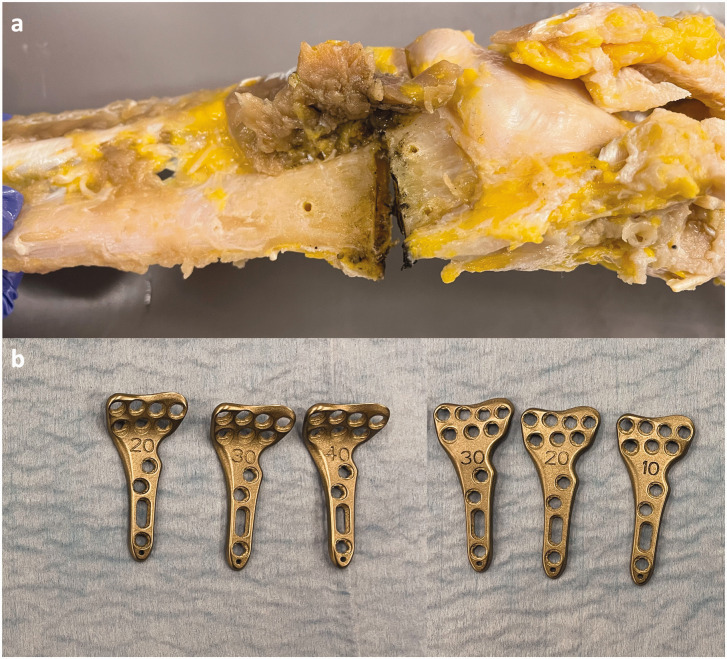
The custom-made 3-D-printed anterior plates used to induce the sagittal deformities of the distal epiphyseal in the palmar (left; 20°, 30° and 40°) and dorsal (right; 10°, 20° and 30°) planes.

The anatomical reference plate was removed, and a double osteotomy was performed at the metaphyseal level, creating a bone-free space of approximately 5 mm (Figure 2(a)). Three palmar (+20°, +30° and +40°) and three dorsal (−10°, −20° and −30°) tilt deformities were simulated successively using six custom-made three-dimensional (3-D) printed anterior plates provided by Newclip Technics™ (3-D models based on the Xpert Wrist 2.4, PSI radius division, Haute-Goulaine, France) (Figure 2(b)). A biomechanical engineer designed the plates using computer-aided design software and the plates were manufactured out of titanium. The parts were tribofinished, sandblasted and cleaned. We used the predefined pilot holes to secure each plate to vary the palmar/dorsal tilt (sagittal plane) without altering the radial tilt and ulnar variance (coronal plane).

### Radiological assessment

Standard radiographs with anteroposterior (AP) and lateral views of all wrists were made before the radial osteotomy and after each deformity induced in the sagittal plane using a mini C-arm. The digital radiographs were viewed on OsiriX^©^ (Pixmeo® 2016, Geneva, Switzerland). Palmar/dorsal tilt, radial tilt (accuracy of 2°) and ulnar variance (accuracy of 0.2 mm) were measured. We checked the match between the sagittal angulation induced by the 3-D-printed plate and the radiographic palmar/dorsal tilt and found no difference for each of the experiments.

### Forearm pronation and supination measurement

Before the radial osteotomy and after each deformity induced in the sagittal plane, an independent examiner (experience Level IV (Tang and Giddins, 2016)) positioned the forearm in maximum unconstrained pronation and supination. Then, the range of motion was measured in degrees (accuracy of 2°) using a goniometer. The palmar cortical surface of the radius, just proximal to the osteotomy, was used as a landmark for positioning the goniometer to standardize each specimen's measurements.

### Statistical analysis

The Shapiro−Wilk test was used to evaluate data normality. As all data sets evaluated were considered non-normally distributed (*p* < 0.001), quantitative data is presented as median and interquartile range (IQR = Q3–Q1). Qualitative variables are described by their counts and percentages The Wilcoxon signed-rank test was applied for between-group comparisons. The significance level was set at *p* < 0.05.

## Results

### Radiographic findings

Before the radial osteotomy, the median (IQR) palmar tilt, radial tilt and ulnar variance were 11° (2°), 18° (2°) and 3 mm (2°), respectively. After the radial osteotomy and plate fixation, there was no significant difference (*p* > 0.05) between the median palmar or dorsal tilt measured on radiographs and the sagittal angulation provided by each 3-D-printed plate. In addition, the radial tilt and ulnar variance values did not change during the entire experiment (*p* > 0.05).

### Forearm pronation and supination outcomes

Before the radial osteotomy, the median (IQR) pronation and supination values were 55° (18°) and 70° (12°), respectively. After the radial osteotomy and plate fixation, there was a significant decrease (*p* < 0.05) in the median pronation and supination values for all palmar and dorsal tilt deformities, except for the supination value with 20° palmar tilt (*p* = 0.18). For this deformity, the degree of pronation was significantly decreased compared with the pre-osteotomy condition (*p* = 0.02).

Pronation was reduced by 18% for 20° and 30° of palmar tilt and 28% for 40° of palmar tilt. The values were reduced by 45% for 10° dorsal tilt, 63% for 20° dorsal tilt and 80% for 30° dorsal tilt. Quantitatively, the loss of pronation amplitude ranged from 10° to 15° for the palmar tilt deformities and 25° to 45° for the dorsal tilt deformities.

Supination was reduced by 7% for 20° palmar tilt, 21% for 30° palmar tilt and 30% for 40° palmar tilt. Values were reduced by 15% for 10° and 20° palmar tilts and 35% for 30° palmar tilt. Quantitatively, the loss of supination amplitude ranged from 5° to 20° for the palmar tilt deformities and from 10° to 25° for the dorsal tilt deformities. A summary of the results is presented in Table 1.

**Table 1.** Outcomes of forearm pronation and supination measurements before radial osteotomy and after each induced deformity.

Experimental condition	Forearm pronation	*p*-value^a^	Forearm supination	*p*-value^b^
*Before radial osteotomy/Baseline*	55° (63°–45°)		70° (75°–63°)	
*After radial osteotomy and 3D printed anterior plate fixation*				
20° Palmar tilt deformity	45° (50°–35°)	**0.02**	65° (70°–58°)	>0.05
30° Palmar tilt deformity	45° (50°–32°)	**0.02**	55° (65°–50°)	**0.04**
40° Palmar tilt deformity	40° (48°–30°)	**0.02**	50° (62°–47°)	**0.02**
10° Dorsal tilt deformity	30° (38°–18°)	**0.02**	60° (65°–50°)	**0.04**
20° Dorsal tilt deformity	20° (33°–15°)	**0.02**	60° (65°–48°)	**0.03**
30° Dorsal tilt deformity	10° (30°–8°)	**0.02**	45° (58°–40°)	**0.02**

Data shown are median and interquartile ranges (IQR). The accuracy of the measurements was 2°. Statistically significant *p*-values shown in bold.

^a^*p*-values are results compared with median pronation value before osteotomy.

^b^*p*-values are results compared with median supination value before osteotomy.

## Discussion

These findings from our study indicated that pronosupination (PS) movement is impacted by both palmar and dorsal tilt deformities, without any radial shortening, even for minimal sagittal angulation. Furthermore, the pronation range was more impaired than the supination range, and dorsal tilt deformities caused the greatest loss in forearm rotation.

Data on the impact of distal radial malunions on PS range of motion vary between studies. Other authors evaluated the impact of distal radius sagittal deformities on the forearm rotation in cadaver studies. Nishikawi et al. (2015) reported that a 20° palmar tilt deformity significantly decreases supination (around 20%) while preserving pronation. They hypothesized that such a deformity tightens the triangular fibrocartilage complex (TFCC) in supination, positioning the ulnar head palmar in relation to the distal radioulnar joint (DRUJ), thus restricting supination only. Kihara et al. (1996) reported that a 30° dorsal tilt deformity affects pronation and supination movements. They also hypothesized that these changes were related to DRUJ incongruency, possibly combined with damage to its soft tissue stabilizers, such as the TFCC, pronator quadratus and interosseous membrane. However, these authors did not find any significant change in pronation and supination for 10° to 20° dorsal tilt deformities. They contend that because severe osseous misalignment was required to produce a significant loss of PS under experimental conditions, soft tissues contracture is likely a primary cause of the reduced PS range of motion found in clinical practice for less severe deformities.

Conversely, Bronstein et al. (2014) noted that dorsal tilt deformities up to 30° had no impact on pronation or supination. In their study, loss of forearm rotation was caused by either ulnar translation of the distal radius (from 5 mm) or radial shortening (from 10 mm). They considered that severe DRUJ incongruency was caused by a frontal deformity, not a sagittal one.

The measurements resulting from our cadaver study are not entirely in line with these authors. We found that from a 10° dorsal tilt or 30° palmar tilt, both pronation and supination decreased significantly. Contrary to Nishikawi et al. (2015), a 20° palmar tilt significantly decreased pronation while supination was minimally affected. Thus, we believe an isolated sagittal displacement of 20° (palmar or dorsal) induces forearm bone misalignment leading to a malposition of the DRUJ and is responsible for loss of both pronation and supination.

Measurements of pronation and supination are frequently used to assess the effectiveness of surgical treatments for distal radial malunion (Pauchard et al., 2016). Graham (1997) proposed radiographic criteria beyond which a corrective radial osteotomy can be discussed to promote optimal functional recovery of the forearm and wrist. He recommends limits of 20° palmar tilt and 15° dorsal tilt. Our results seem to support these recommendations to preserve forearm rotation. Graham also provides additional criteria, such as the radial tilt and the ulnar variance.

Our study has limitations. First, the sample size was small, which did not allow us to study how the induced deformities interact with different anatomical variations and renders our statistical significance susceptible to error. In their cadaver study, using 3-D models of the forearm bones, Daneshvar et al. (2020) concluded that the rotational anatomy of the radius and ulna varies significantly between individuals. However, our results were consistent from one specimen to another. Second, this was a cadaver study, and therefore the results might not be directly transferable to clinical practice, particularly in predicting the effects of rehabilitation on functional recovery. Third, we could not assess the extent to which soft tissue contracture can contribute to the restriction of forearm rotation. Indeed, it was difficult to evaluate the contribution of DRUJ soft tissue stabilizers relative to forearm bone stability. Finally, the distal radial deformities were made in the same sagittal axis. Although the deformity is often classified as palmar or dorsal, extra-articular distal radial malunion is usually a complex 3-D deformity, including sagittal, frontal and rotational deformities. Thus, it is essential to consider the forearm as an interconnected, functioning unit in which an abnormality of these three parameters can affect the forearm's rotation (Athlani et al., 2020). Further studies are required to explore the impact of frontal and rotational deformities on the loss of forearm rotation, especially their respective effects on pronation or supination.
